# Amalgam Mixing

**Published:** 1919-03

**Authors:** 


### AMALGAM MIXING

We may abandon the notion that it is necessary to mix mercury and alloy in definite proportions in order to secure the strongest, and in other ways the best fillings. Provided only that sufficient mercury is used to obtain thorough amalgamation, it matters little how much more is used. The general tendency is to make the mix too dry.

Long trituration in a mortar, or even kneading in the hand, improves the quality of the resulting amalgam. Instead of producing a weaker, or otherwise inferior filling as some writers have claimed, the soft, velvety mix obtained by thorough amalgamation with plenty of mercury produces a stronger, more homogeneous filling that is easier to adapt properly to the walls of the cavity.

By a simple expedient we can easily prevent an amalgam made from a high grade alloy, from setting before we have had time to insert the filling. After triturating thoroughly with sufficient mercury to make a smooth, plastic mix, we have merely to avoid expressing the excess mercury until just before placing in the cavity. Moreover, an amalgam that has already begun to harden somewhat, may be softened again without loss of strength by adding a little more mercury and working the mass until again plastic.

Thousands of carefully controlled experiments have all supported the natural, common-sense view: namely, that any procedure which makes for more intimate union between the mercury and the particles of alloy also makes for better fillings. Thorough trituration with a properly designed mortar and pestle, and sufficient mercury to make sure of wetting every granule of alloy, help in producing the desired intimate union. These same experiments have also proved that the composition of the packed amalgam, that is to say, the proportion of mercury retained in the filling, is practically independent of how much excess mercury is used in triturating, but is governed mainly by the pressure under which the excess mercury has subsequently been expressed, either before or after insertion in the tooth cavity. The greater the pressure employed in condensing, the less will be the percentage of mercury retained, and the stronger will be the resulting filling. Moreover, thorough trituration with sufficient mercury, not only produces an amalgam that is easier to work and a filling that is more homogeneous and stronger, but it accomplishes another desirable end: it gets rid of the expansion that takes place during the early stages of hardening—an expansion that all dental alloys have recently been found to show.

The proportions formerly recommended as correct for Twentieth Century Alloy, namely, five parts by weight of alloy to seven of mercury, or 1.4 times as much mercury as alloy, produces an excellent amalgam that can be worked satisfactorily; but some effort is required to make a smooth mix, and the mass sets more rapidly than some operators find convenient. Increasing the mercury ratio to 1.6, or even to 2.0, makes amalgamation much easier and more complete.

While numerous experiments have repeatedly demonstrated that close adherence to a definite mercury ratio is entirely unnecessary for securing good results with such an alloy as Twentieth Century Perfected, it by no means follows that an alloy and mercury gauge is a superfluity. Quite the contrary: a good measuring device is a time-saver, a material-saver, and a great aid in securing uniformity of results. An efficient gauge will almost instantly drop into the mixing mortar the amount of alloy required for the filling in hand, and at the same time just enough mercury for easily obtaining thorough amalgamation. More than enough is merely waste of material. This saving of time and money means economy. But the uniformity of mix secured is still more important.

In order to secure really thorough amalgamation, trituration with a properly designed mortar and pestle is essential. Mechanical amalgamators are in the main merely mixing devices and, consequently, are far from efficient, in spite of their cost and apparent convenience. Neither can a proper mix be made with the square of rubber dam, the finger cot, etc., or the palm and thumb. Some such grinding action under strong pressure as is so easily obtained with mortar and pestle is essential to bringing about intimate incorporation of mercury and alloy. A moment's subsequent spatulation on a glass slab with a palette knife, while not particularly objectionable, does not seem to offer any special advantage.

The time devoted to amalgamation has a marked influence upon the crushing strength of the filling made from the resulting amalgam. As stated above, a fundamental principle which makes for more intimate union between mercury and alloy also makes for better fillings. Maximum strength is reached only after trituration in a mortar continued as long as four to six minutes. If continued up to ten minutes there is but little change, while if carried on for only three minutes the strength is noticeably less, and it falls off with increasing rapidity as the triturating time is still further shortened. If the amalgam is triturated for only about a minute and a half, subsequent kneading in the hand will, to be sure, bring about a slight increase in strength; but time consumed in kneading is not nearly so effective as the same time spent in mortar trituration, and it does not produce such uniform results. Moreover, from a sanitary standpoint the mortar and pestle are greatly to be preferred, because no epithelium, or skin detritus, is thereby worked into the mass. —*Dental Quarterly*, September, 1917.

